# CE-MRA-FLOWnet: Fast and accurate generative artificial intelligence-based aortic hemodynamic mapping from contrast-enhanced magnetic resonance angiography

**DOI:** 10.1016/j.jocmr.2026.102773

**Published:** 2026-07-07

**Authors:** David Dushfunian, Haben Berhane, Ethan Johnson, Aniket Dehadrai, Michael Markl, Bradley D. Allen

**Affiliations:** a Department of Radiology, Feinberg School of Medicine, Northwestern University, Chicago, Illinois, USA; b Department of Biomedical Engineering, McCormick School of Engineering, Northwestern University, Evanston, Illinois, USA

**Keywords:** AI, contrast-enhanced MRA, 4D flow MRI, generative adversarial networks, hemodynamics, aorta

## Abstract

**Background:**

Four-dimensional (4D) flow magnetic resonance imaging (MRI) hemodynamic biomarkers (e.g., peak velocity and wall shear stress [WSS]) have shown promise for improved risk-stratification in patients with thoracic aortic disease (TAD). However, lengthy 4D flow scan times, complex data analysis and need for dedicated expertise limit clinical translation. In this study, we developed a fluid-physics informed deep generative adversarial neural network, CE-MRA-FLOWnet, to predict aortic hemodynamics directly from standard-of-care contrast-enhanced magnetic resonance angiography (CE-MRA) images.

**Methods:**

We retrospectively identified 1392 patients (age: 52±12years, 1011 male, 954 with bicuspid aortic valve, BAV; 438 with tricuspid aortic valve, TAV) who underwent paired clinical 4D flow MRI and CE-MRA between 2011 and 2020 for suspected TAD. The CE-MRA-FLOWnet used CE-MRA data (1127 for network training, 265 for testing) to generate a prediction of aortic hemodynamics. 4D flow-measured aortic systolic three-dimensional (3D) blood flow velocity vector fields served as ground truth. Analysis included comparison of AI-derived and ground truth aortic systolic peak velocity (PV) and WSS, the relative area of the ascending aorta (AAo, %) exposed to elevated WSS, and aortic valve stenosis severity grading. The predictive value of CE-MRA-FLOWnet hemodynamics metrics was assessed in a subgroup of 133 BAV patients with known multi-year adverse outcomes.

**Results:**

CE-MRA-FLOWnet training time was 8100 min; inference time per CE-MRA was 0.88±0.05 seconds. AI-derived PV showed strong regional agreement and low biases with ground truth 4D flow (0.00–0.04 m/s), and relative differences within 10.6%-12.7%. Aortic WSS also demonstrated minimal bias (-0.01–0.04 Pa) and close alignment of the area of elevated WSS between AI and 4D flow (19.6±15.1% vs. 19.3±16.2%, p=0.84). AS severity was accurately classified in 88% (233/265) of cases, with all grading errors limited to a one-grade difference (Kappa 0.84). Receiver operating characteristic (ROC) analysis showed that CE-MRA-FLOWnet derived hemodynamic metrics outperformed diameter alone for predicting adverse outcomes (PV area under the curve [AUC] = 0.73–0.76; WSS = 0.83–0.88; diameter = 0.52–0.62).

**Conclusion:**

CE-MRA-FLOWnet accurately predicts aortic hemodynamics in TAD patients using standard-of-care CE-MRA images. Our results demonstrate potential for clinical integration by providing physicians with near real-time hemodynamic data from widely available clinical CE-MRA images.

## Graphical abstract

**Figure:**
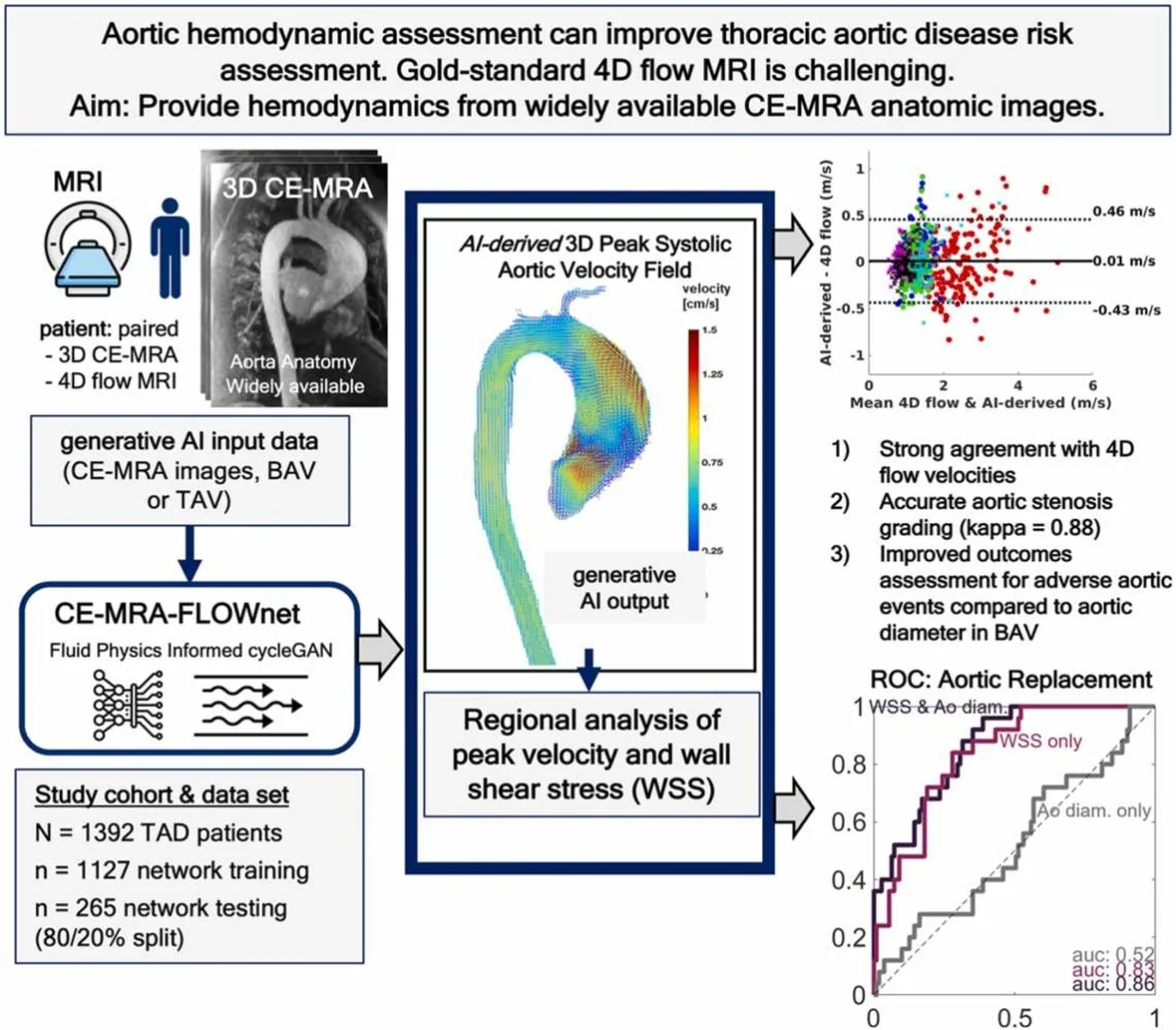

## 1 Introduction

Thoracic aortic disease (TAD) encompasses a spectrum of conditions affecting the thoracic aorta, including progressive dilation, aneurysms, and dissection, which are associated with significant morbidity and mortality. The etiology of TAD is multifactorial, with aortic valve disease being the most prevalent underlying factor, affecting approximately 2%–3% of the U.S. population. However, current risk stratification strategies primarily rely on aortic diameter thresholds, which have limited predictive accuracy for identifying patients at risk of acute aortic complications [1], [2], [3].

Contrast-enhanced MRA (CE-MRA) is widely used in clinical practice for evaluating aortic anatomy and monitoring TAD. While CE-MRA offers critical anatomical insights essential for TAD diagnosis and management, making aortic hemodynamics data available could enhance risk stratification and inform more personalized treatment planning. The emergence of four-dimensional (4D) flow MRI has enabled *in vivo* assessment of complex aortic three-dimensional (3D) hemodynamic in the thoracic aorta [4], [5], [6], [7], [8], [9], [10], [11], [12], [13]. Growing evidence suggests that altered 3D blood flow patterns associated with aortic valve disease can play a key role in driving aortic dilatation and promoting adverse remodeling of the aortic wall [14], [15], [16], [17], [18], [19], [20], [21], [22]. Studies have demonstrated that abnormal aortic valve morphology—such as bicuspid aortic valve (BAV) and aortic valve stenosis (AS)—can induce abnormal blood flow patterns and elevated wall shear stress (WSS) in the ascending aorta (AAo), factors that are associated with progressive aortic dilatation and development of TAD [7], [14], [23], [24], [25]. Although recent advances in magnetic resonance imaging (MRI) acceleration techniques have reduced 4D flow MRI acquisition times to approximately 5–10 min [26], its broader clinical adoption remains limited by lengthy scan durations, labor-intensive post-processing, and specialized expertise required for data interpretation.

We have recently demonstrated that advances in artificial intelligence (AI), specifically using deep generative adversarial networks (GANs) for image-to-image translation, can be harnessed to derive blood flow information from anatomical data [27]. In that study, a fluid physics–informed cycle-consistent GAN (FPI-CycleGAN) was successfully used to derive accurate 3D aorta hemodynamics in under one second, using a 3D segmentation of the thoracic aorta as network input data. The deep learning model showed strong agreement with aortic 4D flow MRI, the established reference standard, particularly in predicted aortic peak systolic velocities and classification of the severity of AS.

However, the study had notable limitations. It required extensive preprocessing, including the generation of a 3D aortic segmentation as network input data. In addition, the input was derived from 4D flow MRI rather than more widely used clinical anatomic imaging of the chest such as CE-MRA. Because CE-MRA images were not included in the AI model training set, the application of the FPI-CycleGAN approach was limited to MRI studies which included 4D flow MRI, and it did not allow for more widespread application in standard clinical exams in the setting of TAD.

The aim of the present study was to overcome these limitations by developing a novel fluid physics–informed deep generative model (CE-MRA-FLOWnet), trained and validated on a large TAD cohort with paired CE-MRA and 4D flow MRI. In this improved approach, we sought to expand the clinical applicability of our technique by using the network to derive thoracic aorta 3D hemodynamics directly from standard clinical CE-MRA images as the only input data. This updated network requires no additional imaging or aorta segmentation to perform quantification. Compared to prior work, we conducted a more comprehensive evaluation of AI performance for predicting aortic WSS using a previously introduced WSS heatmap framework [19], [28], [29], which enables identification of regions with elevated WSS by comparison to age- and sex-matched control populations. We also investigated the influence of demographic and physiological factors - including age, blood pressure, heart rate, and aortic dimensions—on the AI model’s predictive performance. Finally, we evaluated the predictive value of CE-MRA-FLOWnet-derived hemodynamic metrics in a subgroup of BAV patients with known multi-year adverse outcomes.

## 2 Methods

The data that support the findings of this study are available from the corresponding author upon reasonable request.

### 2.1 Study cohort

Patients for this study were retrospectively identified from a database of patients who underwent standard-of-care cardiothoracic MRI, including 4D flow MRI and 3D CE-MRA of the thoracic aorta, at a tertiary academic institution between 2011 and 2020. Inclusion criteria were referral for clinical imaging for suspected thoracic aortic disease and assessment of aortic dimensions or aortic valve abnormalities. Aortic valve status (BAV or tricuspid aortic valve, TAV), maximum diameter of the ascending aorta, body mass index (BMI), systolic and diastolic blood pressure, and heart rate were derived from the medical records. As shown in Fig. 1, exclusion criteria were unavailable or non-standard 4D flow imaging protocols not suitable for standard 4D flow analysis, unavailable paired CE-MRA data, or insufficient 4D flow data (poor data quality, severe velocity aliasing). Of note, 82% (1141/1392) of subjects who were ultimately included for this study were also included in our previously published work [27]. We believe this overlap is justified because the current study focuses on a completely different approach for deriving velocity fields from CE-MRA images rather than aortic 3D segmentations from 4D flow as was done in the prior work. Moreover, as described below, these subjects were distributed into training and testing groups randomly so the reported results by group are completely unique.

**Fig. 1:**
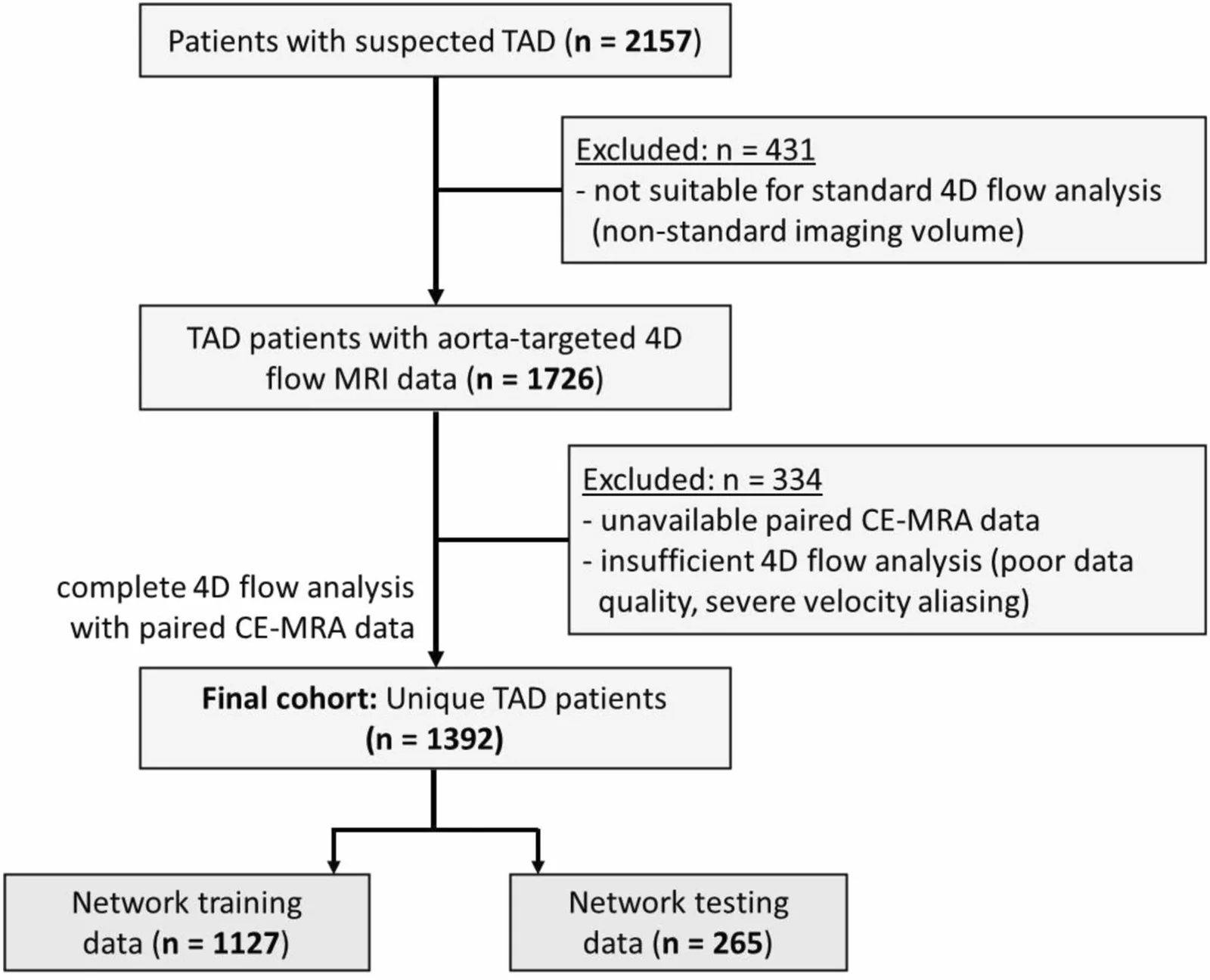
Study enrollment flow chart. Of the unique 2157 patients with suspected thoracic aorta disease (TAD), 1392 had paired 4D flow MRI and contrast-enhanced magnetic resonance angiography (CE-MRA) data of the thoracic aorta. Training and testing of the CE-MRA-FLOWnet was performed based on a random 80%/20% split of the final study cohort of 1127 datasets for network training and 265 for network testing, respectively. *4D* four-dimensional

In addition, the study cohort included n=136 healthy controls across a wide range of ages and equally divided for sex (age range 19–81 years, 67 male, 69 female) with no known cardiovascular disease and a normally functioning TAV who underwent a research MRI including 4D flow MRI scans. The control 4D flow data were used to derive a normal population WSS distribution and compute age and sex specific regionally resolved 95% confidence intervals for physiologically normal aortic WSS.

Informed consent was obtained from all controls. All patients undergoing standard-of-care MRI were enrolled by retrospective chart review and waiver of consent. All subjects were included in the study according to procedures approved by the Northwestern University Institutional Review Board (IRB). Institutional IRB reference numbers are STU00219441 and STU00067816.

### 2.2 MRI Imaging—4D flow MRI and CE-MRA of the thoracic aorta

All subjects underwent cardiothoracic MRI using either 1.5T (Aera, Avanto, or Espree, Siemens Healthineers, Erlangen, Germany) or 3T MRI systems (Skyra, Siemens Healthineers, Erlangen, Germany). 4D flow MRI data were acquired in a sagittal-oblique 3D imaging volume with 3D coverage of the thoracic aorta during free-breathing with respiratory navigator and electrocardiographic (ECG) gating. 4D flow MRI pulse sequence parameters were as follows: spatial resolution = 1.8–4.0 mm^3^; velocity encoding = 150–500 cm/s, temporal resolution = 32.8–44.8 ms. 3D CE-MRA data were acquired in sagittal-oblique orientation (spatial resolution: 0.59–2.3 mm^3^) following standard-of-care administration of contrast agent: Gadavist, Magnevist (Bayer Healthcare, Berlin, Germany); Multihance (Bracco Diagnostic, Cranbury, New Jersey); Ablavar (Lantheus Medical Imaging, Billerica, Massachusetts). The velocity encoding (venc) for each 4D flow MRI was informed by a phase contrast venc scout image with three-chamber and left ventricular outflow tract (LVOT) coverage. 4D flow MRI was acquired after 3D CE-MRA imaging at the end of the same MRI examination.

### 2.3 4D flow MRI data analysis & ground truth data for network training and resting

All 4D flow MRI scans underwent standard 4D flow pre-processing using an in-house developed workflow programmed in MATLAB (Mathworks, Natick, Massachusetts) as previously described [30], [31], [32], [33]. Briefly, 4D flow MRI data were corrected for eddy current background phase offset errors, Maxwell terms, and velocity aliasing. Next, a time-averaged 3D phase contrast angiogram (PC-MRA) was calculated and used as input to generate a 3D segmentation of the thoracic aorta using a previously developed convolutional neural network (CNN) [34]. The aortic 3D segmentation was used to mask blood flow velocities inside the thoracic aorta. Peak systole was defined as the cardiac time frame with the highest velocity averaged over the entire aortic volume. The 4D flow measure peak systolic 3D velocity vectors field (v_x_,v_y_,v_z_) served as the ground truth data for training and testing the deep learning CE-MRA-FLOWnet (see Fig. 2).

**Fig. 2:**
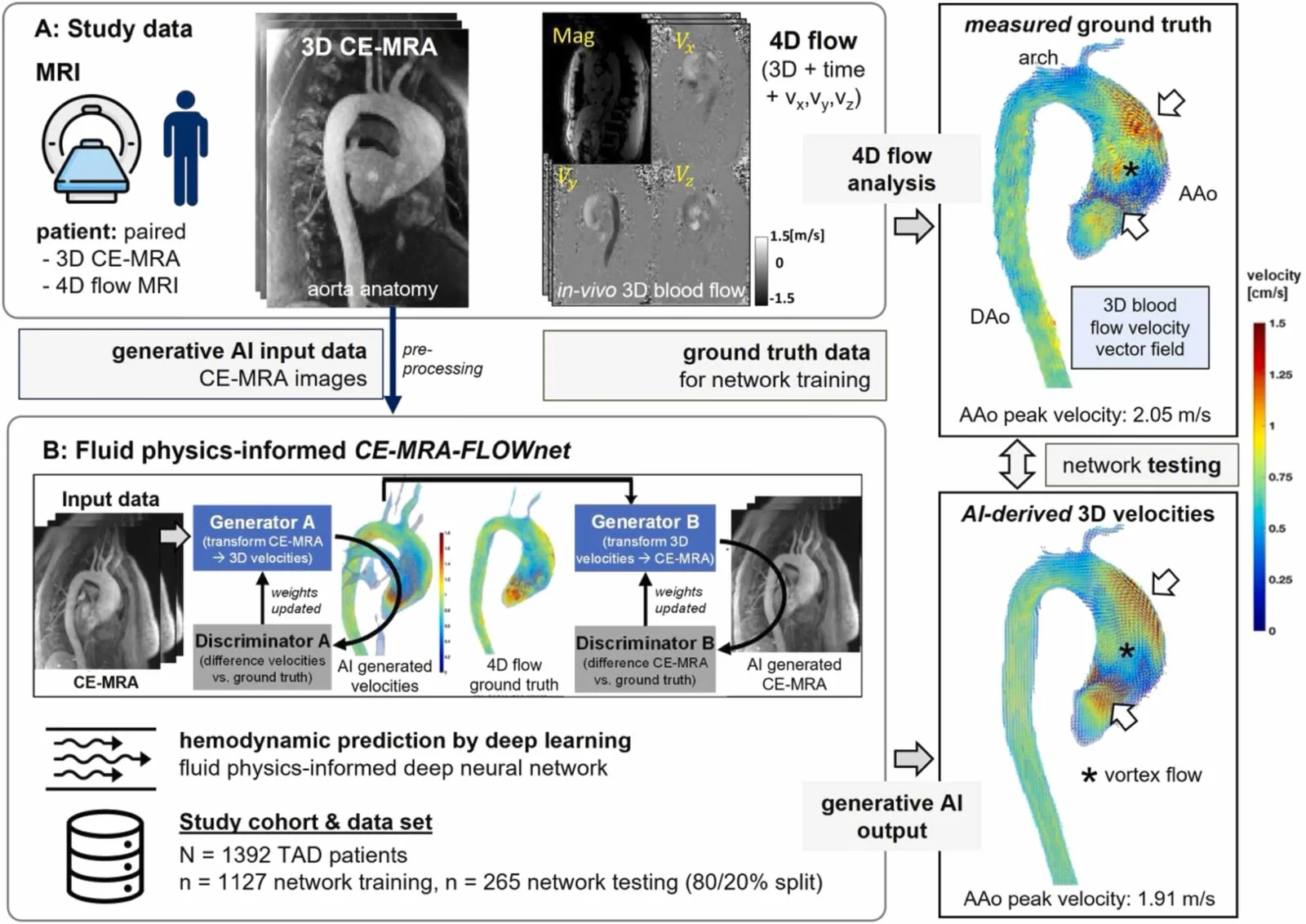
Illustration of the data flow for AI-driven prediction of aortic hemodynamics from CE-MRA images using the fluid physics-informed CE-MRA-FLOWnet. **A:** Paired contrast CE-MRA and 4D flow MRI data were acquired for each patient. 4D flow MRI-derived systolic 3D velocity vector fields served as ground-truth data for CE-MRA-FLOWnet training and testing. **B:** Standard-of-care CE-MRA images and valve type (BAV or TAV) were used as the only input to the CE-MRA-FLOWnet to predict systolic 3D velocity vector fields. Comparison of AI-derived vs. 4D flow measured results (right) show close agreement in aortic 3D velocity patterns such as valve jet flow along the aortic wall (white arrows) and vortex flow at the mid-AAo. *AAo* ascending aorta, *arch* aortic arch, *DAo* descending aorta, *CE-MRA* contrast-enhanced MRA, *TAD* thoracic aortic disease

### 2.4 Deep learning—network architecture

The data flow for network training and testing of the CE-MRA-FLOWnet is shown in Fig. 2. The CycleGAN architecture was based on our previously developed and validated deep learning network [27] and was re-trained for 3D CE-MRA input data. The 3D CE-MRA DICOM data covering the thoracic aorta was used as the input data and the systolic 3D blood flow velocity vector field measured by 4D flow MRI served as ground truth data. During training, the CE-MRA data was registered to the 4D flow MRI without any additional processing. For registration, the thoracic aorta was segmented from CE-MRA and a rigid registration was applied between the CE-MRA segmentation to the PC-MRA-based segmentation from the corresponding 4D Flow MRI. The transformation matrix was then applied to the non-segmented CE-MRA data set. To assess adequacy of registration, we calculated the dice score between the aortic segmentation of the CE-MRA and the 4D flow MRI and values less than 0.50 were treated as failures. If the first attempt at automated registration between CE-MRA and 4D flow MRI failed, manual adjustment was made to better align the data. For testing, the CE-MRA data was downsampled to a resolution of 2.5×2.5×3 mm, with no registration performed. This reflects an advance from our previous approach which required 3D segmentation as input. Any additional processing (like segmentation) was performed after the systolic velocity vector fields were generated from the network.

The CycleGAN was composed of two generators and two discriminators. The generator consisted of a 3D hybrid CNN based on a Densenet/Unet while the discriminator was composed of 5 convolution layers with 4×4 kernel size, followed by batch normalization and a leaky ReLU layer [33], [34]. The first 4 convolution layers had increasing filter sizes (64,128, 256, and 512) with the final layer having a filter size=1. The architecture of the DenseNet/Unet is a CNN that has an encode-decoder design of a conventional Unet with DenseNet blocks [33], [34]. In this new development, we have removed the final 1 channel convolution layer and softmax layer and adding a 3-channel convolution layer. Hyperparameters were determined and optimized in prior work [27]. We chose to use the same hyperparameters in the current development due to the similar architecture and loss functions.

During network training, Generator A derived 4D hemodynamics from the CE-MRA images and Discriminator A was used to distinguish between the AI-derived and 4D flow MRI ground truth velocity vector field data. The AI-derived 4D hemodynamics data were then passed on to Generator B to re-derive the input (3D CE-MRA images), while Discriminator B was used to distinguish between the AI-estimated and original input CE-MRA images. In summary, there were 2 generators (A and B) and 2 discriminators (A and B) being trained:

• Generator A learned to transform input CE-MRA images to 3D velocity vector fields.
• Generator B learned to transform 3D velocity vector fields back to CE-MRA images.
• Discriminator A learned to differentiate between ground truth 3D velocity vector fields and generated 3D velocity vector fields.
• Discriminator B learned to differentiate between the original input CE-MRA images and generated CE-MRA images.

Similar to previously reported strategies, for the Discriminators and Generators the loss functions utilized cycle-consistency loss and fluid physics-informed loss functions in our deep learning model [27], [35]. These concepts included exploiting the well-known properties of conservation of mass and blood as an incompressible fluid, which requires 3D velocity vector fields to meet ‘divergence-free’ and ‘flow momentum balanced’ conditions. These conditions were mathematically expressed according to Navier Stokes equations and integrated in the CE-MRA-FLOWnet loss function. For the conservation of the mass, we performed the mean-squared difference of the velocity gradient for the CNN output to reproduce the divergence of the 4D flow ground-truth. Additionally, we calculated the material derivative from the velocity vector fields for both the AI-derived and 4D flow MRI velocity vector fields and performed the mean squared error between them to incorporate it into the loss function. Because CE-MRA_FLOWnet only calculates a single time point, only spatial, but not temporal, derivatives were computed. The network architecture design included loss functions for minimizing both the differences in velocity magnitude between AI-derived and ground truth *in-vivo* velocity vector fields. We adjusted the weighting of each component of the loss function to ensure each component has the same order of magnitude. While cycleGANs are designed for unpaired image translation, we incorporated these supervised-learning-based loss functions through the mean-squared error of the material derivative and velocity gradient to improve accuracy of the velocities generated by the network. If unpaired data were used, our fluid-physics-based loss functions would likely fail to converge only be dependent on the cycleGAN-based losses. As shown in Fig. 2**,** the CE-MRA-FLOWnet output was a CE-MRA-derived 3D velocity vector field (v_x_,v_y_,v_z_) at peak systole.

### 2.5 Deep learning - training and testing

The CE-MRA-FLOWnet was trained on a randomly selected 80% of datasets, while testing was performed on the remaining 20% of the study cohort. Of note, subjects had to have aortic stenosis severity classification available for inclusion in the testing cohort. For network training, 4D flow MRI and CE-MRA were spatially registered and the CE-MRA data were down-sampled to the 4D flow MRI resolution. Separate networks were trained for BAV and TAV with the 80% training and 20% testing ratio in both cohorts. The criteria for early stoppage were if the improvement in the loss function was less than 1e-4 over 25 epochs, indicating that the training has plateaued. CE-MRA images were the only input for the network and a priori knowledge of valve type was required for training and testing the appropriate network. For testing, the CE-MRA inputs remained spatially un-registered and underwent pre-processing down-sampling to the same resolution as the 4D flow ground-truth. Training and testing were programmed in TensorFlow 2 with an ADAM optimizer and an initial learning rate of 2e^−4^ and performed on a workstation equipped with an i9–10900K processor and a single RTX 3090 graphical processing unit (GPU).

### 2.6 Hemodynamic quantification

Both ground truth in-vivo 4D flow MRI and AI-derived 3D velocity vector fields underwent the same analysis pipeline for hemodynamic quantification (Fig. 3). This included the calculation of a systolic velocity maximal intensity projection (MIP) [36] and derivation of 3D WSS distribution along the surface of the aorta at peak systole as described previously [29]. Regional peak systolic velocities (top 5%) were calculated in 3D regions of interest covering the ascending aorta (AAo), aortic arch, and descending aorta (DAo).

**Fig. 3:**
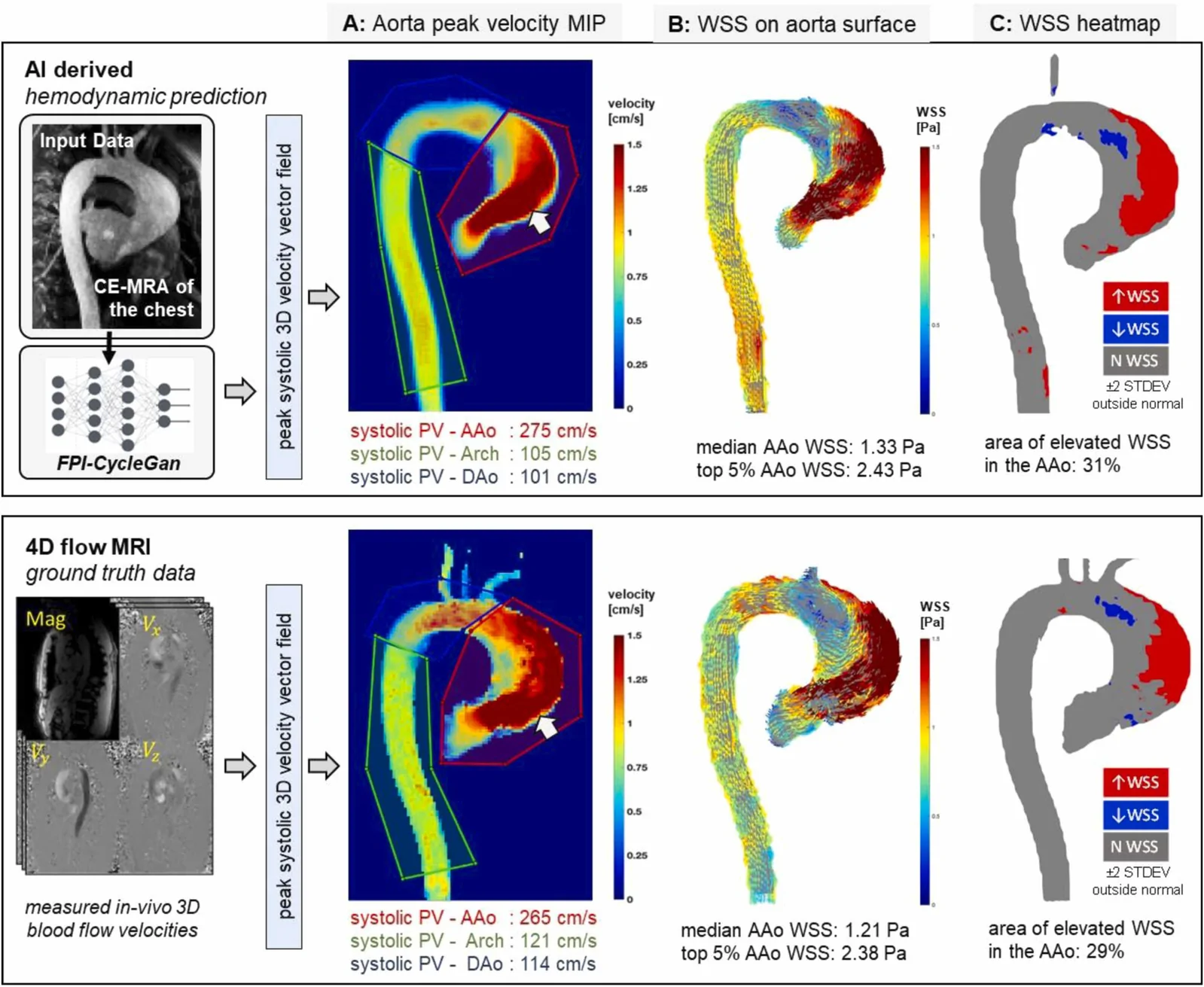
Side-by-side comparison of AI-derived (**top**) and 4D flow measured (**bottom**) peak systolic velocity maximum intensity projection (MIP, **A**), wall shear stress (WSS) mapped on the aorta surface (**B**), and patient-specific WSS heatmaps (**C**) in an example patient with bicuspid (BAV) and mild aortic valve stenosis (AS) severity grade. All hemodynamic maps (**A, B, and C**) were derived from peak systolic 3D velocity vector fields using a standardized hemodynamic analysis workflow. Patient-specific WSS heatmaps were calculated by comparing the patient's WSS distribution to and age and sex matched healthy control population to identify areas of elevated WSS (WSS outside the 95% confidence interval of the control population WSS distribution, red areas in **C**). Marked changes in aortic velocities and WSS (high velocity valve jet patterns, white arrows) were well reproduced by the AI model. *AAo* ascending aorta, *Arch* aortic arch, *DAo* descending aorta

Data analysis also included the calculation of patient-specific WSS heatmaps relative to population-averaged WSS maps of age- and sex-matched healthy controls (Fig. 3**, right**), as reported previously [19], [28], [29]. Briefly, WSS magnitude on the segmented aortic surface was calculated at peak systole. Median and maximum WSS (top 5%) for the AAo were computed for each patient. To identify regions of abnormal WSS, the WSS distribution for a normal control population was created for each patient using 4D flow-derived systolic WSS magnitude data from 10 or more age- and sex-matched healthy controls. A personalized patient WSS heatmap was generated by spatial co-registration of the corresponding population-average WSS distribution to the patient aorta segmentation. Regions with elevated WSS outside the 95% confidence intervals of the age- and sex-matched healthy controls (visualized as red areas mapped on the aorta 3D geometry, Fig. 3 **right**) were categorized as abnormal and visualized on the 3D aorta surface. The relative area (%) of the AAo exposed to elevated WSS was quantified. All 4D flow MRI-derived hemodynamics data were considered the ground-truth for testing of the CE-MRA-FLOWnet performance.

### 2.7 Aortic valve stenosis grading

AS severity grades were based on measured peak velocities and defined as follows: none (peak velocity ≤ 2.5 m/s), mild (2.5 < peak velocity ≤ 3.0 m/s), moderate (3.0 < peak velocity ≤ 4.0 m/s), and severe (peak velocity > 4.0 m/s).

### 2.8 Diagnostic value of AI-derived hemodynamics for outcomes assessment in BAV

An institutional database was used to access outcomes of BAV subjects included in our testing cohort. The electronic health record was queried to collate outcomes, specifically ascending aorta replacement, aortic valve replacement, or aortic dissection. Patients with prior aortic surgery were excluded. Also, patients with outcomes occurring any time before or a limited time after imaging were excluded (surgery: 6 months, dissection: 1 month), as it was possible that imaging in this interval was specifically performed to evaluate the outcome of interest.

### 2.9 Statistical analysis

We used *t*-tests (or non-parametric equivalent as necessary) to compare AI-derived and measured regional peak velocities and WSS measures, and to compare CE-MRA-derived hemodynamics between outcome groups. Bland-Altman analysis was used to calculate bias and limits of agreement (LOA) between CE-MRA-FLOWnet-derived and 4D flow-derived ground-truth hemodynamic data. To evaluate CE-MRA-FLOWnet performance for predicting AS severity grade and outcomes, a Receiver-Operator Curve (ROC) was created and the area under the curve (AUC) was calculated. In addition, Cohen's Kappa score and Matthews Correlation Coefficient (MCC) were calculated to assess the agreement with AS severity derived from measured in-vivo 4D flow MRI data. For the BAV outcome cohort, the CE-MRA-FLOWnet estimated AAo peak systolic velocities and maximum WSS and maximum aorta diameter were used to fit linear models for prediction of the outcome as a binomial variable (stable vs. intervened). For each hemodynamic parameter (peak systolic velocity, maximum WSS), two linear models were created, including or excluding aorta diameter as a variable. Additionally, a model using only diameters and age was also derived. The same analysis was then performed for 4D flow MRI-derived AAo peak systolic velocities and maximum WSS. The predictive performance of the linear models was evaluated using ROC analysis with AUC as a performance metric.

### 2.10 Impact of demographic, physiologic and anatomic parameters on AI model performance

To assess whether accounting for demographic (age, BMI), physiologic (heart rate, blood pressure, aortic regurgitant volume and percentage, cardiac output), or anatomic parameters (maximum aortic diameter) could improve AI performance, we performed a series of univariate correlation analyses to test for association between these parameters and the observed AI error (relative difference of AI-derived vs. 4D flow ground truth peak velocities). Statistically significant associations (p<0.05) were identified and used to build a multivariate AI correction model. The multi-variate model with the highest R^2^ was used to correct CE-MRA-FLOWnet peak velocity estimations and AS severity grading. Bland-Altman analysis and Cohen's Kappa score (corrected AI-derived vs. ground-truth) were calculated to test for possible improved agreement with ground truth data.

## 3 Results

### 3.1 Study cohort

A total of 2157 patients were identified in our single-center database of clinical cardiothoracic MRI exams. Of these, 431 patients (20%) were excluded due to the 4D flow MRI protocol being not suitable for standard analysis (non-standard field-of-view orientation). Additionally, 320 patients (15%) were excluded due to unavailable paired CE-MRA data. Finally, 14 patients (0.5%) were excluded due to insufficient 4D flow data quality (gating/motion artefacts or severe velocity aliasing). After exclusions, a total of 1392 patients (age range: 18–91, 52 ± 15 years, 1011 male) remained for final inclusion in the study cohort (Fig. 1). Of the 1392 patients, 954 (69%) presented with congenitally abnormal BAV and 438 (31%) had regular TAV morphology. Study cohort demographics are summarized in Table 1.

**Table 1:** Study cohort demographics

**Cohorts**	**Network training**	**Network testing**	**p-value**
**N**	1127	265	-
**Age [years]**	52±16	51±15	0.6
**BAV/TAV**	774/353	180/85	0.8
**Sex**	819 males	192 males	0.9
**Weight [kg]**	84.0±17.7	86.2±18.0	0.1
**Height [m]**	1.76±0.11	1.76±0.10	0.3
**BMI [kg/m**^**2**^**]**	27.1±5.0	27.7±4.8	0.1
**AS severity grade**			0.7
**None**	819	191	
**Mild**	84	31	
**Moderate**	101	35	
**Severe**	116	8	
**Missing AS grade**	7	0	-

Values are expressed as mean and standard deviation. *BAV* bicuspid aortic valve*, TAV* tricuspid aortic valve*, BMI* body mass index*, AS* aortic valve stenosis

### 3.2 Deep learning network training and testing

Training and testing of the CE-MRA-FLOWnet was performed using the final cohort of 1392 patients (network training: n=1127 [80%, n = 774 BAV and n = 353 TAV], network validation and testing: n = 265 [20%, n = 180 BAV and n = 85 TAV]). There were no significant differences in patient demographics or AS severity grades between training and testing cohorts (Table 1). Of the 1127 training datasets, 109 datasets required manual adjustments due to poor registration. Network training was conducted over a total of 400 epochs and the total training time was 8100 min (see Supplemental Figure 5 for training and validation loss over epochs). Once the training was concluded, the inference time for network execution on testing was 0.88±0.05 s per CE-MRA input data for each patient. The pre-processing time on average was 9.72 +/- 2.59 s. This combined with the inference time led to a total analysis time of 10.91 +/- 0.52 s per case in the testing cohort.

### 3.3 Network performance—hemodynamic quantification

A comparison of AI-derived vs 4D flow measured aortic hemodynamics for a representative case is shown in Fig. 3. The individual images represent systolic peak velocity maximum intensity projections (MIPs, Fig. 3**A**), WSS mapped on the aorta surface (Fig. 3**B**), and patient-specific WSS heatmap (Fig. 3**B**) of the thoracic aorta. All hemodynamic maps were calculated from the AI-derived and 4D flow MRI-measured systolic 3D velocity vector fields. Side-by-side comparisons of aortic velocity and WSS patterns derived by the CE-MRA-FLOWnet with those measured by 4D flow MRI show close agreement for velocity ranges and distinct elevated regions of WSS patterns across the aorta. Please see the Supplement for extensive planar analysis of flows and velocities across in the ascending aorta, aortic arch, and descending aorta.

These results are corroborated by findings across the entire testing cohort (n=265) for AI-derived vs. measured (4D flow) metrics of aortic hemodynamics which are summarized in Table 2 **and** Fig. 4. Bland-Altman comparisons demonstrated low non-significant bias (0.00–0.04 m/s) and good limits of agreement (LOA: ±0.36 to ±0.57 m/s) between AI-derived and 4D flow measured regional aortic peak velocities across the testing datasets. The only significant bias in systolic peak velocity quantification was found for the aortic arch (AI: 1.08±0.26 m/s; 4D flow: 1.12±0.31 m/s, bias: 0.04 m/s, p=0.01). Nonetheless, average relative differences between AI-derived and ground truth in-vivo peak velocities were low and ranged between 10.6% and 12.7%. Given nearly equivalent standard deviations between 4D flow measured and AI-derived hemodynamic parameters in Table 2, there are no findings to suggest GAN mode collapse. Please see Supplemental Table 2 for a breakdown of performance by aorta valve-morphology. Supplemental Figure 6 shows a comparison of network performance with and without the physics-informed loss function.

**Table 2:** Peak velocities and WSS measures (average ± standard deviation) for the AAo, aortic arch, DAo in the test set of n=265 patients

	**Peak velocities [m/s]**			**Median WSS [Pa]**			**Top 5% WSS [Pa]**			**WSS heatmap area [%]**
	**AAo**	**arch**	**DAo**	**AAo**	**arch**	**DAo**	**AAo**	**arch**	**DAo**	
***In-vivo*****measured**	2.15±0.84	1.12±0.31	1.13±0.26	1.03±0.3	0.74±0.23	0.78±0.23	1.91±0.67	1.10±034	0.97±0.25	19.6±15.1
**AI-derived**	2.16±0.77	1.08±0.26	1.12±0.22	1.05±0.28	0.79±0.20	0.84±0.19	1.91±0.62	1.12±0.29	1.00±0.22	19.3±16.2
**p-value**	0.95	0.08	0.78	0.46	0.02*	0.01*	0.91	0.37	0.13	0.84
**Bland-Altman Analysis AI-derived vs. in-vivo hemodynamics**										
**bias±LOA**	0.0±0.57	-0.04±0.36	-0.01±0.38	0.03±0.29	0.04±0.22	0.04±0.24	-0.01±0.57	0.01±0.27	0.02±0.27	-0.28±9.7
**p-value**	0.81	0.01*	0.62	0.06	0.02*	0.01*	0.82	0.13	0.18	0.39
**% difference**	10.6±8.6	11.9±10.5	12.7±11.3	11.9±10.3	13.7±12.7	15.3±13.6	10.3±9.4	11.2±8.6	11.6±10.0	11.3 [3.1–24.9]

Bland-Altman analysis demonstrated close agreement between AI-derived and ground-truth hemodynamic measures with relative differences between ranging between 10.3% and 15.3%. The only significant differences were found for peak velocities in the aortic arch and median WSS in the arch and DAo. *Indicates significant differences between AI-derived and in-vivo measured. % difference was calculated as the mean relative difference of AI-derived vs. 4D flow measured metrics. Values for WSS heatmap area % differences are reported as median and confidence intervals due to non-normal distribution. *WSS* wall shear stress, *LOA* limits of agreement, *AAo* ascending aorta, *DAo* descending aorta

**Fig. 4:**
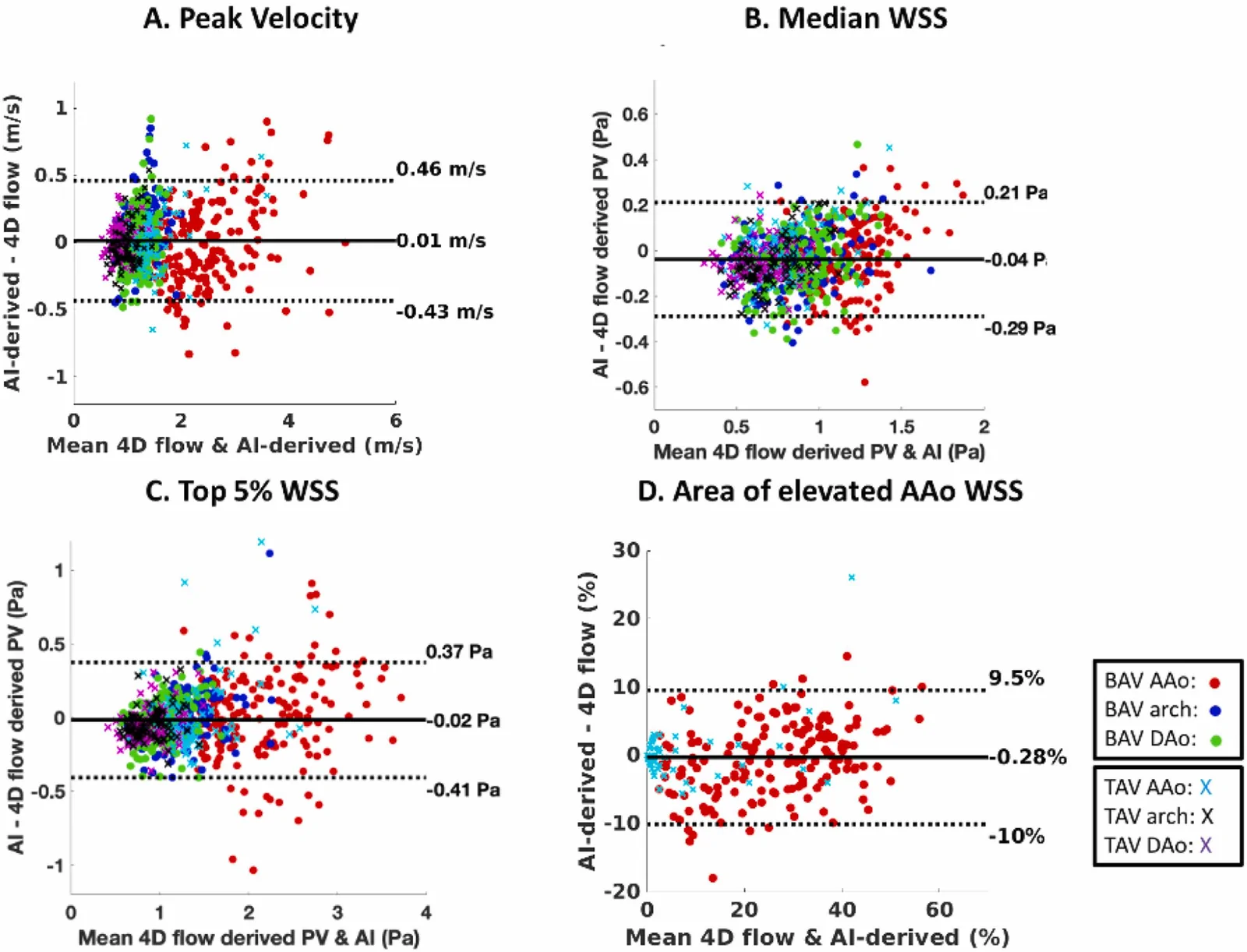
Bland-Altman plots comparing AI-derived and in-vivo 4D flow MRI-based regional aortic peak velocities, medium and maximum WSS as well as the detected area of elevated WSS on heatmaps. Patients with BAV and TAV are indicated by different data symbols (o and x). Low biases for all comparisons demonstrate good to excellent agreement between AI-derived and in-vivo 4D flow MRI-based velocity and WSS quantification. *AAo* ascending aorta, *arch* aortic arch, *DAo* descending aorta, *WSS* wall shear stress, *3D* three-dimensional, *4D* four-dimensional, *BAV* bicuspid aortic valve, *TAV* tricuspid aortic valve

For WSS quantification, no significant differences were observed for the maximum (top 5%) WSS across all aortic regions (bias: −0.01 to 0.02 Pa, p>0.13). For median WSS, the aortic arch and descending aorta ascending aorta showed small but significant biases (arch: AI: 0.79±0.20 Pa, 4D flow: 0.74±0.23 Pa, bias: 0.04 Pa, p=0.02; DAo: AI: 0.84±0.19 Pa, 4D flow: 0.78±0.23 Pa, bias: 0.04 Pa, p=0.01). The average relative differences for both median and maximum WSS were below 16% (10.3%–15.3%) Areas of elevated WSS on heatmaps in the AAo were similar between ground-truth and CE-MRA-FLOWnet estimates (19.6±15.1% vs. 19.3±16.2%, p=0.84), with low bias (bias: 0.3%, LOA: ±9.7%, p=0.39) and mean relative difference compared to the 4D flow ground truth data of 11.3%.

### 3.4 Network performance—AS grading

AI-derived estimates of velocities accurately identified AS severity grade (none, mild, moderate, and severe) in 233 of 265 (88%) of patients. Any discrepancies in AS severity grade were limited to a maximum of one grade difference from the ground truth stenosis grade. As shown in Fig. 5, ROC curves demonstrated good to excellent performance for AI-based AS classification (AUC=0.80–0.98). The Kappa score for the full cohort was 0.84 [95%CI: 0.74–0.93]. The MCC for the entire test set was 0.746, and by grade: none = 0.841; mild = 0.535; moderate = 0.775; Severe = 0.796. These findings indicate strong to excellent agreement between AI-derived and 4D flow ground-truth aortic valve stenosis grading. We found that misclassified patients tended to have higher ascending aorta and arch velocities as well as higher aortic regurgitant volumes/fractions and cardiac output. See Supplemental Table 3 for groupwise comparison by aortic valve stenosis grade and between group comparisons of classified/misclassified subjects and Supplemental Table 4 for a comparison by stenosis grade.

**Fig. 5:**
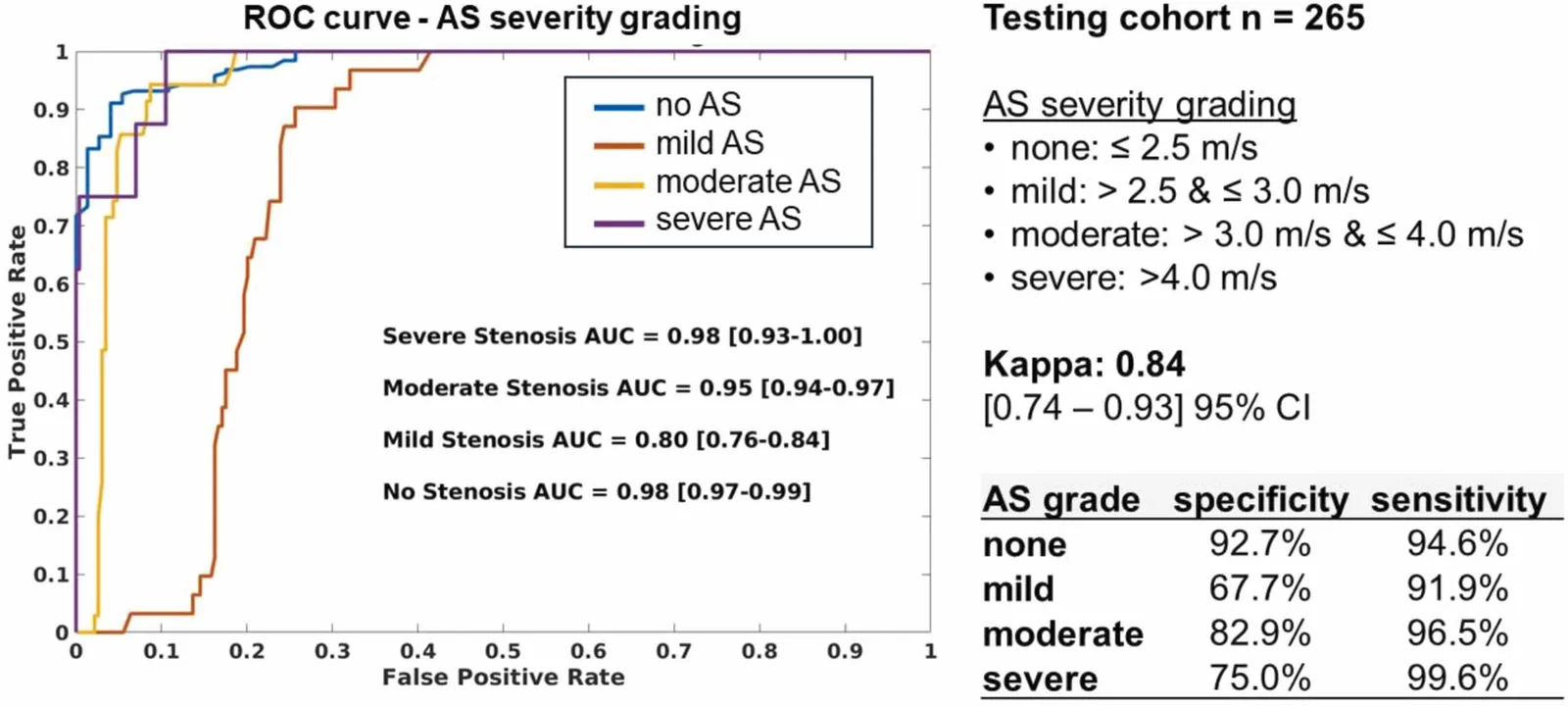
Severity grading of aortic valve stenosis determined by in-vivo 4D flow MRI (ground truth) vs. AI-derived hemodynamic quantification from CE-MRA input data. The ROC across the entire testing cohort shows good agreement between both methods (AUC = 0.80–0.98), supported by Kappa 0.84 indicating excellent agreement. *AS* aortic valve stenosis, *ROC* receiver operator curve

### 3.5 Network performance—BAV outcomes

A total of n = 133 BAV patients of the CE-MRA-FLOWnet testing set were eligible for inclusion in the outcomes analysis. Of this group, 100 subjects had no outcome, 26 subjects had AAo replacement (including 2 subject who experience a type A aortic dissection), and 7 subjects had isolated AV replacement. Group characteristics, including mean follow-up duration for the 3 groups (7.2–9.0 years), are presented in Supplemental Table 1. As shown in Supplemental Figure 3A, CE-MRA-FLOWnet derived peak velocity in the no outcome group (2.2 ± 0.5 m/s) were lower than both the ascending aorta replacement (2.7 ± 0.6 m/s, p =.003) and AV replacement (2.6 ± 0.6 m/s, p = 0.002) groups. There was no difference in two surgery subgroups (p = 0.9). Similarly, for WSS the no outcome group (2.3 ± 0.6 Pa) was lower than both the ascending aorta replacement (2.8 ± 0.7 Pa, p = 0.009) and AV replacement (2.6 ± 0.7, p = 0.03). There was no difference in two surgery subgroups (p = 0.9). Similar results were also seen with ground-truth 4D flow MRI (Supplemental Figure 3B). As shown in Fig. 6**,** ROC analysis found that both peak systolic velocity (AUC=0.73–0.76) and WSS (AUC=0.83–0.86) derived from CE-MRA-FLOWnet were better predictors of progression to adverse outcomes than baseline aortic diameter alone (AUC=0.52–0.62). Best performance was achieved for models combining hemodynamic metrics with maximum aortic diameter (peak systolic velocity: AUC=0.73–0.96, maximum WSS: AUC = 0.86–0.97). Similar performance was achieved with 4D flow MRI for peak systolic velocity (AUC = 0.73–0.88) and WSS (AUC = 0.76–0.88). Combined models using 4D flow MRI hemodynamics and aortic diameter showed overall high performance as well (peak systolic velocity: AUC=0.80–0.97, maximum WSS: AUC = 0.81–0.95).

**Fig. 6:**
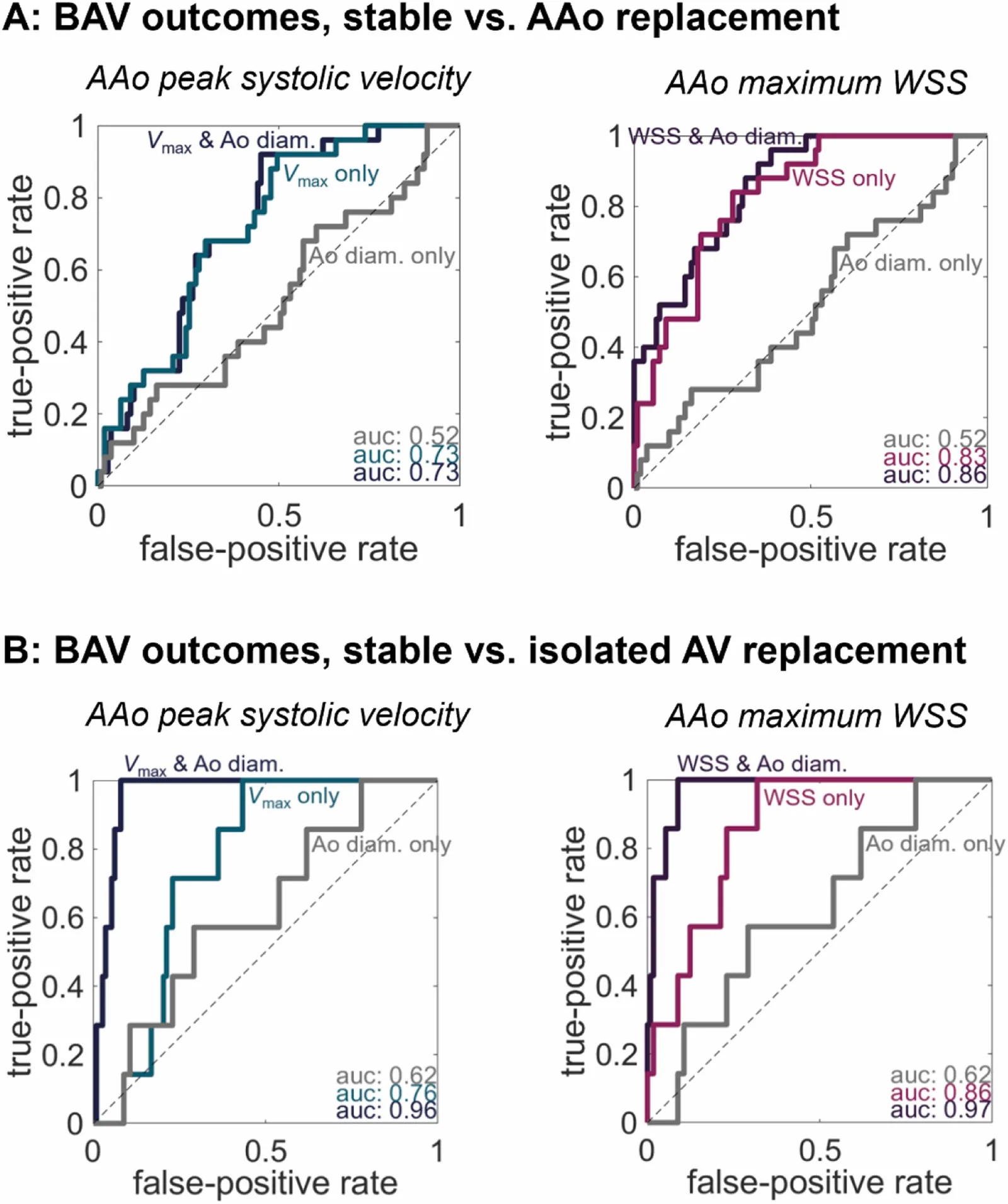
ROC analysis for classifying BAV patients who had stable outcomes vs. AAo replacement (A) and isolated AV replacement (B) using peak systolic velocity and maximum WSS derived from CE-MRA-FLOWnet - relative to baseline maximum aortic diameter (Ao diam.) alone. *AAo* ascending aorta, *WSS* wall shear stress, *BAV* bicuspid aortic va, *ROC* receiver operating characteristic

### 3.6 Network performance—impact of demographic, physiological, and anatomic factors

As summarized in Table 3, the testing cohort included a wide range of demographic (age, sex, BMI), physiologic (blood pressure [BP], heart rate, aortic regurgitant volume and fraction, cardiac output), aortic dimensions, and scan parameters (voxel size and field-of-view [FOV]). Among the variables, univariate correlation analysis revealed weak but significant associations between AI errors and systolic blood pressure (r=0.14, p=0.02), age (r=0.17, p<0.01), body mass index (r=0.13, p=0.04), and aortic regurgitant volume (r=−0.15, p=0.02). Diastolic blood pressure, heart rate, maximum aortic diameter, sex, aortic regurgitant fraction, cardiac output, voxel size, and FOV had no impact on AI results. Additionally, we saw no significant difference between in the AI error and the scanner field strength (p=0.20). Multivariate correlation analysis revealed that the following model was most closely associated (adjusted R^2^ = 0.048, p = 0.004) with AI error:

AI error = AI-derived systolic PV + 0.002 systolic BP + 0.003 age + 0.005 BMI - 0.48.

**Table 3:** Results for univariate correlation analysis of AI model error (AI-derived vs. ground truth peak systolic velocity) with testing cohort characteristics age, sex, BP, HR, BMI, maximum (max) diameter of the AAo, AR volume and fraction, and cardiac output

	age [years]	sex [male / female]	systolic BP [mmHg]	diast. BP [mmHg]	BMI [kg / m^2^]	HR [bpm]	max AAo diam. [mm]	AR Vol (mL)	AR Fraction (%)	CO (L/min)	Voxel size (mm)	FOV (mm)
mean ±std / N	51.9±14.7	192 / 73	125.1±16.8	74.5±10.1	27.8±5.0	67.8±5.3	40±6.67	49±29	33±14	9.4±2.9	1.1±0.1, 1.1±0.1, 1.5±0.2	389.0±31.2, 298.1±26.7, 45.3±6.53
(min, max)	(18, 79)	-	(84, 235)	(45, 100)	(16.7, 44.4)	(44, 122)	(23, 61)	(4, 170)	(4, 95)	(3.9, 23.8)	(0.6,1.3), (0.6,1.3), (1.1,2.2)	190,470), (214,400), (33,60)
correlation with PV error	**age [years]**	**sex [male / female]**	**systolic BP [mmHg]**	**diast. BP [mmHg]**	**BMI** **[kg / m**^**2**^**]**	**HR [bpm]**	**max AAo diam. [mm]**	**AR Vol (mL)**	**AR Fraction (%)**	**CO (L/min)**	**Voxel size** **(mm)**	**FOV** **(mm)**
r	**0.17**	0.99	**0.14**	0.04	**0.13**	0.03	0.06	**-0.15**	0.12	0.13	0.05	0.01
p-value	< 0.01*	0.99	0.02*	0.49	0.04*	0.6	0.39	0.02*	0.06	0.05	0.49	0.83

*BP* blood pressure, *HR* heart rate, *BMI* body mass index, *AAo* ascending aorta, *AR* aortic regurgitant. * and bold type indicate significant relationships

The model was applied across the entire testing cohort to analyze the effect of correcting for influences of systolic BP, age, or BMI upon possible AI errors. Repeat Bland-Altman analysis between AI-derived and 4D flow-measured aortic peak velocities after correction (Supplemental Figure 1) demonstrated only very minor changes (bias±LOA pre-correction: 0.01±0.44 m/s; post-correction: 0.00±0.43 m/s). In addition, network performance for AS severity grading was similar (AUC = 0.80–0.98) and maintained an excellent Kappa score (0.82 [0.71–0.92], Supplemental Figure 2). Of note, we found that a model which included aortic regurgitant volume was also statistically significant but had a lower correlation with AI error (adjusted R^2^ = 0.045, p = 0.004). Corrected aortic peak velocities again showed minimal differences (bias±LOA: 0.00±0.55) and there was no impact on AS classification (Kappa = 0.82). These findings indicate that demographic, physiological, and anatomic factors had a minimal impact on AI network performance.

## 4 Discussion

In this study, we built on our prior work in using AI for the prediction of aortic hemodynamics as an alternative to 4D flow MRI by training and validating a fluid-physics informed deep generative adversarial neural network (CE-MRA-FLOWnet) with clinical standard-of-care CE-MRA images and valve morphology phenotype (BAV or TAV) as the only input data. The main findings of the study are 1) the deep generative model enabled direct estimation of 3D thoracic aorta hemodynamics from CE-MRA images alone, eliminating the need of a separate 4D flow MRI data acquisition or aorta 3D segmentation as network input data; 2) peak velocity, WSS, and AS stenosis severity derived from AI-predicted velocity fields demonstrated good to excellent agreement those from with 4D flow MRI, the established reference standard; 3) demographic and physiological factors or aortic dimensions had minimal impact on AI performance; 4) CE-MRA-FLOWnet derived hemodynamic metrics outperformed diameter alone for predicting adverse multi-year outcomes in a subgroup of BAV patients; and 5) hemodynamic output estimation could be achieved in under one second, followed by standardized hemodynamic visualization and quantification.

Evaluation of aortic hemodynamics is increasingly recognized as playing an important role in understanding cardiovascular pathophysiology and assessing patient risk. 4D flow MRI has emerged as a promising diagnostic tool, offering detailed quantification of in-vivo, 3D blood flow dynamics throughout the cardiac cycle [4], [5], [6], [7], [8], [9], [10], [11], [12], [13]. However, its broader clinical adoption remains limited by the need for specialized hardware, technical expertise, and lengthy acquisition times. Our findings highlight the potential of deep generative networks to rapidly derive 4D flow–like hemodynamic information from widely available CE-MRA thoracic aorta scans, which require only ∼30 s of scan time and a single patient breath hold. This approach may allow for broader clinical integration, enabling more accessible and actionable hemodynamic insights for physicians.

Our results demonstrate that the hemodynamic measures obtained from purely anatomical images with CE-MRA-FLOWnet provided accurate prediction of systolic peak velocity, WSS, and AS severity grade. Among the tested parameters, only peak velocity in the aortic arch and median WSS in the arch and DAo were significantly different between AI estimates and ground-truth 4D flow MRI data. Nonetheless, the observed difference remained close to an acceptable range of 10%–16%, which corresponds to previously reported inter-observer variability for 4D flow MRI analysis [30], [37].

In the outcomes analysis of 133 BAV patients, CE-MRA-FLOWnet-derived hemodynamic biomarkers demonstrated significant associations with the progression to surgical intervention. Both peak systolic velocity and maximum WSS were elevated in patients who underwent ascending aortic or aortic valve replacement compared to those without adverse outcomes, consistent with prior reports linking abnormal flow patterns to aortopathy progression [19], [20]. ROC analysis underscored the prognostic value of peak velocity and WSS, which outperformed current guideline-recommended measures [2], maximum aortic diameter, in predicting adverse outcomes. Notably, predictive accuracy was highest when hemodynamic metrics were combined with aorta diameter. These findings highlight the potential of CE-MRA-FLOWnet-derived biomarkers to augment traditional morphologic assessment and improve risk stratification in BAV aortopathy.

Other studies have introduced and investigated the use of deep learning for the estimation of aortic blood flow. A recent study by Lin et al. [38] reported on the development of deep neural networks for rapid estimation of aortic blood flow features in the thoracic aorta—velocity, pressure, and WSS. In contrast to the present study, their work was based on synthetic aortic geometries derived from statistical shape modeling of 40 healthy aortas as network input data. Model testing showed low average errors (∼3% for velocity, ∼5% for WSS). A similar approach was employed by PajazitiI et al. [39] in a study which aimed to replicate conventional aortic computational fluid dynamic (CFD) simulation of aortic hemodynamics using machine learning for faster and automated predictions. Using 3000 simulations on synthetic 3D aortic shapes derived from real patient data (n=67), the machine learning models achieved average errors of 4% for velocity on 200 test shapes. We speculate that better network performance compared to our study (10%–16% for peak velocities and WSS) stems from network training and testing that was based on hemodynamic ground truth data provided by CFD simulations assuming laminar flow, rather than true in-vivo 4D flow MRI measurements used in our study. Another study by Sun et al [40] developed and evaluated a deep learning method to predict time-varying in-plane 2D blood flow velocities within the heart from long-axis cine MRI data, using 4D flow MRI as ground truth data. Compared to our findings, the model achieved a similar high correlation with in-vivo flow measurements but resulted in substantial blood velocity underestimation up to 27%, possibly related to the smaller study cohort (78 patients, 34 controls) used for training and testing of the deep learning model.

We believe the current development represents a fundamental leap forward from our prior work which focused on deriving hemodynamics from an input mask that was generated directly from 4D flow MRI [27]. While that method demonstrated the principle that aorta hemodynamics could be quantified from anatomic input using deep learning, the current method represents a significant practical advance demonstrating 1) the method works on data derived from other sources besides 4D flow MRI (in this case CE-MRA), and 2) no a priori anatomic segmentation is required for strong performance by the network. While these developments may seem relatively minor from a deep learning perspective, the significance for potential clinical implementation is critical. The ability for quantification to be performed from standard CE-MRA with minimal user interaction/segmentation can significantly broaden the reach of aorta hemodynamic quantification in patient care. Moreover, while the model could be trained for specific tasks such as accurate aortic stenosis grading, the ability to provide full velocity vector field quantification that allows for regional hemodynamic assessment and WSS quantification is essential to provide novel targets for risk assessment in TAD.

Compared to our own previous work [27], which demonstrated the initial feasibility of AI-derived aortic hemodynamics, agreement here with ground truth data measured by 4D flow MRI was similar but mildly decreased (10%–16% vs. 6%–10% for peak velocity and WSS). Notably, CE-MRA-FLOWnet only required CE-MRA images as input and a priori knowledge of valve morphology (BAV vs. TAV). As we have shown, age, systolic blood pressure, BMI, and aortic regurgitation volume correlated with peak velocity error. However, using a multivariate model to correct for these covariates resulted in no significant change in peak velocity quantification. Thus, given these variables’ limited impact on prediction accuracy and with the goal of balancing accuracy and clinical feasibility, we felt there was no justification for retraining the model with a larger number of inputs. Moreover, given these findings, we speculate observed differences can be attributed to directly using CE-MRA images as network input data compared to 4D flow-derived 3D aorta segmentations in our prior work, i.e., network input data that was inherently more closely aligned with the ground truth data. Interestingly, in AS classification, misclassified subjects had higher peak velocities, higher degree of aortic regurgitation, and higher cardiac output, which suggests concomitant aortic regurgitation may impact this assessment even though it does not significantly impact overall metrics of velocity quantification. While we have assessed model performance relative to several technical, demographic, and clinical factors, there may be other unknown significant contributors to estimation accuracy that would only become apparent with systematic evaluation of data from new sites, new acquisition protocols, or new patient profiles. This represents an area of future investigation. Nonetheless, the ability to derive full systolic 3D aortic hemodynamics directly from CE-MRA images without pre-processing represents a major advance and may allow for broader clinical adoption while maintaining acceptable hemodynamic predictions with uncertainties in the range of 10–16%. Future studies should include a sensitivity analysis to determine the possible impact on these hemodynamic estimation uncertainties on diagnosis and patient management.

## 5 Limitations

This study has several limitations. Generalizability is a common concern for AI-based models, which rely on the size and representativeness of their training data. Our model was trained and tested using data from a single center, which may limit its applicability to datasets acquired from other institutions or using different MRI systems or protocols. Future studies are thus warranted to evaluate multi-site performance. Moreover, we had to exclude 765 potentially eligible subjects from the final testing and training cohort primarily due to inadequate 4D flow data or lack of paired CE-MRA. These exclusions further highlight the inherent challenges associated with 4D flow MRI and the potential of our technique to expand hemodynamic assessment, but may result in selection bias within our final cohort. There is also a relatively high number of AS patients (∼28%) in our cohort which is likely due to the retrospective cohort design which included subjects undergoing CMR and a large number of BAV patients. Our AI model generated 3D velocity vector fields only at peak systole. We incorporated a “physics-informed” loss function for network training, but these principles were not strictly enforced in the final inference. Using these physics-informed losses, the network was trained to generate 4D flow-like peak systolic vector fields and will be subject to the same assumptions (or biases) inherent in the 4D flow MRI. While peak systolic velocity and WSS hold substantial clinical relevance, this approach limits the ability to compute other clinically important parameters that require data across the entire cardiac cycle, such as aortic regurgitant flow and net flow. This approach also limits our ability to provide internal checks of the conservation of mass principle to further validate the AI output. For WSS, this approach is also subject to the same challenges associated with 4D flow MRI WSS quantification, specifically vessel wall definition and accuracy of velocities near the boundary. Additionally, separate networks were used for BAV and TAV requiring *a priori* knowledge of valve morphology. Because we did not have access to aortic valve area, left ventricular functional parameters or both in the majority of testing subjects, we could not consider performance in patients with low flow low gradient AS or assess the impact of LV remodeling and function on performance. We only considered a cycleGAN approach for our network because, even in the registered training data set, there are still portions of the images where the data is not perfectly aligned, both in and outside of the aorta. As such, a supervised network, expecting a precise voxel-wise correspondence between the input and the ground-truth would likely have not performed as well but this was not explicitly evaluated. Moreover, because the CE-MRA and 4D flow MRI images included in the testing data set were not registered, it was not possible to perform voxel-wise comparisons of the predicted velocities to ground truth. Finally, we did not explore the impact of training cohort size or input data type such as native CE-MRA, aortic segmentation, or other data transformations on network performance. This input data analysis remains an important area for future study.

In conclusion, CE-MRA-FLOWnet demonstrated strong performance in predicting aortic hemodynamics in TAD patients using only standard-of-care CE-MRA images and valve type (BAV or TAV) as inputs. These findings underscore the model’s promise for clinical adoption, offering physicians near real-time access to hemodynamic information derived from routine imaging. However, broader validation is essential. Future studies should assess the model’s generalizability across multiple sites and explore its utility in developing novel TAD risk stratification tools through retrospective analysis of hemodynamic metrics in large CE-MRA datasets with established clinical outcomes.

## Disclosures

Ethan Johnson: Receives salary from Third Coast Dynamics. Michael Markl: Ownership or investment interest and fiduciary role in Third Coast Dynamics; Intellectual property interests directly related to this work. Bradley Allen: Ownership or investment interest and fiduciary role in Third Coast Dynamics; Intellectual property interests directly related to this work; Speaking, consulting, or providing educational programs for Siemens Healthineers AG, Circle Cardiovascular Imaging, MRI Online; Travel from Siemens Healthineers AG; Compensation for medical record consultation and/or expert witness testimony; Grant Support from Guerbet. The remaining authors declare that they have no known competing financial interests or personal relationships that could have appeared to influence the work reported in this paper.

## Author contributions

**David Dushfunian:** Writing – review & editing, Writing – original draft, Methodology, Investigation, Formal analysis, Data curation, Conceptualization. **Haben Berhane:** Writing – review & editing, Validation, Software, Project administration, Methodology, Investigation, Formal analysis, Conceptualization. **Ethan Johnson:** Writing – review & editing, Validation, Project administration, Methodology, Investigation, Formal analysis, Data curation. **Aniket Dehadrai:** Writing – review & editing, Investigation, Formal analysis, Data curation. **Michael Markl:** Writing – review & editing, Writing – original draft, Visualization, Validation, Supervision, Software, Resources, Project administration, Methodology, Investigation, Funding acquisition, Formal analysis, Conceptualization. **Bradley D. Allen:** Writing – review & editing, Writing – original draft, Visualization, Validation, Supervision, Software, Resources, Project administration, Methodology, Investigation, Funding acquisition, Formal analysis, Data curation, Conceptualization.

## Declaration of competing interests

The authors declare the following financial interests/personal relationships which may be considered as potential competing interests: Bradley D Allen reports financial support was provided by National Institutes of Health; American Roentgen Ray Society. If there are other authors, they declare that they have no known competing financial interests or personal relationships that could have appeared to influence the work reported in this paper.

## Footnotes

Appendix A Supplementary data associated with this article can be found in the online version at doi:10.1016/j.jocmr.2026.102773.
