# Supplementary material for: CE-MRA-FLOWnet: Fast and accurate generative artificial intelligence-based aortic hemodynamic mapping from contrast-enhanced magnetic resonance angiography
Source: J Cardiovasc Magn Reson. 2026 Jul 7;28(2):102773. doi: 10.1016/j.jocmr.2026.102773 (PMC13755473; doi:10.1016/j.jocmr.2026.102773)
Supplement: Supplementary file 1 [file mmc1.docx]

**supplemental MATERIALs**

**Supplemental Figure 1:** Bland-Altman analysis comparing AI-derived and 4D flow (ground-truth) derived regional velocities after multi-variate correction for age, systolic blood pressure, and body mass index. Patients with bicuspid aortic valve (BAV) and tricuspid aortic valve (TAV) are indicated by different data symbols (o and x). AAo, ascending aorta; DAo, descending aorta; BAV, bicuspid aortic valve; TAV, tricuspid aortic valve; LOA, limit of agreement.

**Supplemental Figure 2:** Receiver Operator Curves (ROCs) for severity grading of aortic valve stenosis determined by in-vivo 4D flow MRI (ground truth) vs. AI-derived hemodynamic quantification from CE-MRA input data, after multi-variate correction for systolic blood pressure, age, and body mass index. Kappa 0.82 indicates excellent agreement. AS: aortic valve stenosis.

**Supplemental Table 1: Baseline characteristics of 133 bicuspid aortic valve patients included in the outcome analysis. AVR – aortic valve replacement.**

**Supplemental Figure 3:** Comparison of *CE-MRA-FLOWnet* (A) and 4D flow MRI (B) derived peak velocity (V_max_) and wall shear stress (WSS) in 133 bicuspid aortic valve patients with known outcomes.

**Supplemental Figure 4:** ROC analysis demonstrating the improved performance for correctly classifying BAV patients who underwent aortic valve replacement using peak velocity (V_max_) and wall shear stress (WSS) derived from CE-MRA-FLOWnet relative to baseline aortic diameter (Ao diam.) alone.

**Supplemental Table 2: Outputs by valve morphology (BAV and TAV) for CE-MRA-FLOWnet which requires a priori knowledge of valve morphology. We also trained and tested a network which did not require knowledge of valve morphology (Combined Cohort Network) which showed reasonable performance but did demonstrate slightly higher biases and limits of agreements relative the valve morphology specific networks.**

|  |  | Correctly Classified | Misclassified | p-value |
| --- | --- | --- | --- | --- |
| Demographics | age (y) | 51.7 ± 14.6 | 54.3 ± 15.2 | 0.35 |
|  | Sex (M/F) | 166/67 | 25/7 | 0.42 |
|  | Systolic BP (mmHg) | 124.6 ± 16.8 | 129 ± 15.9 | 0.16 |
|  | Diastolic BP (mmHg) | 74.8 ± 10 | 72.9 ± 10.7 | 0.32 |
|  | Heart Rate (bmp) | 68.2 ± 12.4 | 65.5 ± 14 | 0.29 |
|  | Height (in) | 68.5 ± 5.5 | 69.6 ± 4.2 | 0.29 |
|  | Weight (lbs) | 185.6 ± 39.9 | 195.6 ± 43.7 | 0.19 |
|  | BMI (kg/m2) | 27.8 ± 5.1 | 28.2 ± 4.3 | 0.65 |
| Aortic Dimensions | mid-AAo (mm) | 39.7 ± 6.9 | 42 ± 6.5 | 0.09 |
|  | SoV(mm) | 41.1 ± 5.7 | 40.9 ± 4.6 | 0.88 |
| CE-MRA-FLOWnet Peak Velocity (m/s) | **Ascending Aorta** | **2.1 ± 0.8** | **2.8 ± 0.4** | **0.000002** |
|  | **Arch** | **1.1 ± 0.3** | **1.2 ± 0.3** | **0.01** |
|  | Desending Aorta | 1.1 ± 0.2 | 1.2 ± 0.2 | 0.18 |
| 4D flow Ground Truth Peak Velocity (m/s) | **Ascending Aorta** | **2.1 ± 0.9** | **2.7 ± 0.6** | **0.0001** |
|  | **Arch** | **1.1 ± 0.3** | **1.3 ± 0.3** | **0.01** |
|  | Desending Aorta | 1.1 ± 0.3 | 1.2 ± 0.3 | 0.12 |
| Additional Hemodynamics | **AR volume** | **46.2 ± 28** | **66.2 ± 29.5** | **0.0004** |
|  | **AR Fraction (%)** | **32.7 ± 15.1** | **39.3 ± 12.6** | **0.02** |
|  | **CO (L/beat)** | **9.1 ± 2.7** | **11.2 ± 3.5** | **0.001** |

**Supplemental Table 3:** Comparison of correct vs. misclassifed aortic stenosis subjects. Misclassified patients tended to have higher peak velocities, higher degree of aortic regurgitation (AR), and higher cardiac output (CO).

|  | CE-MRA Peak Velocity (m/s) | 4D flow MRI Peak Velocity (m/s) | p-value | Bias (LOA) |
| --- | --- | --- | --- | --- |
| None | 1.78 ± 0.43 | 1.72 ± 0.4 | 0.0004 | -0.06 (-0.5,0.4) |
| Mild | 2.7 ± 0.28 | 2.7 ± 0.14 | 0.98 | -0.00 (-0.6,0.6) |
| Moderate | 3.25 ± 0.34 | 3.47 ± 0.26 | 0.0001 | 0.22 (-0.4,0.8) |
| Severe | 4.23 ± 0.71 | 4.59 ± 0.46 | 0.1 | 0.36 (-0.7,1.4) |

**Supplemental Table 4:** Comparing ascending aorta peak velocity quantification between valve stenosis grades in the testing cohort. While there was some statistically significant differences between CE-MRA and 4D flow, the bias and limits of agreement (LOA) were relatively low. These difference were most pronounced in the moderate and severe subgroups but should be interpreted with caution given the relatively low number of patients in these subgroups.

**Supplemental Figure 5:** Training and validation loss over epochs. we did observe divergence between the training and validation loss. While this is indicative of overfitting, however based on our testing dataset, we found that the overfitting was not detrimental. Rather, this is likely due to the network learning the stationary tissue early on, and plateauing when the voxel-wise average is calculated in the validation loss over the 3D volume. We found that with further training the jet flow trajectories and peak velocities showed better agreement with more granular improvement not necessarily captured in the validation loss. This is highlighted in the percentage differences between the AI and the ground-truth as shown in Table 2.

**Supplemental Figure 6:** Comparison of velocity agreement between physics informed and non-physics informed network. The implantation of the physics informed loss function improves correlations between the convolutional neural network (CNN) and 4D flow MRI ground truth. The image qualitatively demonstrate this improved performance as well.

**Supplemental Methods: 2D Regional Planar Analysis**

*Methods*

Planes were placed along the aortic centerline for both 4D flow MRI and CE-MRA-FLOWnet peak systolic vector fields. All forward/net/reverse flows and planar velocity vectors interpolated onto standardized grid of 201 points from 0 to 3, with aortic subregions registered to the grid as follows:

- start of centerline to first innominate branch: rescaled [ 0 , 1 ]
- proximal first innominate branch to distal left subclavian: rescaled [ 1 , 1.5 ]
- distal left subclavian to end of centerline in thoracic aorta: rescaled ( 1.5 , 3 ]

Forward flow is defined as flow with positive dot product to the planar normal vector (centerline tangent).Reverse flow is defined as flow with negative dot product to the planar normal vector. Angular distance is defined as cosine of the vector dot product divided by vector magnitude product. Vector magnitude is defined as square-root sum of squares of vector components. Reference vector magnitudes in angle vs. magnitude comparisons are from 4D flow MRI. All flow values are peak-systolic only, i.e. from one time-point, and as such there is no conservation of mass between planes.

**Supplemental Figure 7:** Planar comparison approach.

*Results*

The overall agreement between CE-MRA-FLOWnet predicted velocities and 4D flow MRI-measured velocities was not markedly different between regions, however, there were certainly nominal differences in the profiles between regions. In particular, the ascending region did have the largest range of flow values, and as such the widest absolute limits of agreement for flow metrics. However, the general distributions of angular error in each region was not notably different by region (see supplemental materials), nor were the distributions of angular error vs. mean velocity magnitude. Correlation coefficients for net and forward flow tended to be higher in the arch and descending regions than in the ascending region (0.88–0.89 vs. 0.81), while they were notably higher for reverse flow in the ascending region than in the arch and descending (0.82 vs. 0.48–0.56). Some of this discrepancy may be attributed to challenges of registering spatial locations across region, and some may be resultant from the overall low quantities of peak-systolic reverse flow observed in the arch and descending regions.

**Supplemental Figure 8A:** Planar Flow and Velocity Vector Comparisons in the Ascending Aorta.

**Supplemental Figure 8B:** Planar Flow and Velocity Vector Comparisons in the Aortic Arch.

**Supplemental Figure 8C:** Planar Flow and Velocity Vector Comparisons in the Descending Aorta.
